# A maskless synthesis of TiO_2_-nanofiber-based hierarchical structures for solid-state dye-sensitized solar cells with improved performance

**DOI:** 10.1186/1556-276X-9-14

**Published:** 2014-01-10

**Authors:** Dharani Sabba, Shweta Agarwala, Stevin S Pramana, Subodh Mhaisalkar

**Affiliations:** 1 School of Materials Science and Engineering, Nanyang Technological University, 639798, Singapore; 2 Energy Research Institute at NTU (ERI@N), Research Techno Plaza, Nanyang Technological University, 50 Nanyang Drive, 637553, Singapore

**Keywords:** Electrospinning, Nanofibers, Hydrothermal, Hierarchical, Solid-state dye-sensitized solar cells, Dye loading, Charge recombination

## Abstract

TiO_2_ hierarchical nanostructures with secondary growth have been successfully synthesized on electrospun nanofibers via surfactant-free hydrothermal route. The effect of hydrothermal reaction time on the secondary nanostructures has been studied. The synthesized nanostructures comprise electrospun nanofibers which are polycrystalline with anatase phase and have single crystalline, rutile TiO_2_ nanorod-like structures growing on them. These secondary nanostructures have a preferential growth direction [110]. UV–vis spectroscopy measurements point to better dye loading capability and incident photon to current conversion efficiency spectra show enhanced light harvesting of the synthesized hierarchical structures. Concomitantly, the dye molecules act as spacers between the conduction band electrons of TiO_2_ and holes in the hole transporting medium, i.e., spiro-OMeTAD and thus enhance open circuit voltage. The charge transport and recombination effects are characterized by electrochemical impedance spectroscopy measurements. As a result of improved light harvesting, dye loading, and reduced recombination losses, the hierarchical nanofibers yield 2.14% electrochemical conversion efficiency which is 50% higher than the efficiency obtained by plain nanofibers.

## Background

Porous material systems are attractive for dye-sensitized solar cells (DSC) as they provide tunable pore size and highly specific surface area with additional advantage of molecular sieving effect and high reactivity. Solid-state dye-sensitized solar cells (ssDSCs) are now emerging as technological and scientific interests by virtue of their stability against corrosion and solvent leakage, which is prevalent in the case of dye-sensitized solar cells employing liquid electrolyte. In spite of the advantages of the ssDSC over liquid DSC, the ssDSC exhibited an initial electrochemical conversion efficiency of 0.74% [1], in which focused research efforts climbed to 7.1% [2]. A critical factor governing the performance of a ssDSC is a good contact between TiO_2_ surface, sensitizer molecule, and hole transporting material (HTM). Proper infiltration of HTM throughout the mesoporous TiO_2_ film is important for a good performing solar cell. This step requires the films to be either highly porous or be very thin (<3 μm). In fabricating porous systems, TiO_2_ nanoparticles have been widely used [3,4]. Although TiO_2_ nanoparticles have high surface area for the attachment of the dye molecules, structural disorders and grain boundary effects lead to the scattering of free electrons and reduction of carrier mobility [5]. In recent times, one-dimensional (1D) nanomaterials have demonstrated distinctive advantage for the energy conversion applications. 1D nanostructures have been studied to improve electron mobility and transport rate [6,7]. However, 1D nanostructures suffer from inefficient dye loading owing to their low surface area. Thus, additional scattering centers are needed on 1D nanostructures to improve light harvesting. Nevertheless, only few studies have reported the synthesis of 1D TiO_2_ nanomaterials because of the high reactivity of hydrolysis and condensation of titanium precursor [8]. Therefore, a careful synthetic strategy is required to fabricate 1D crystalline TiO_2_ materials, which is still a challenge. Secondly, when the films are thin, the performance of the ssDSC cell is hampered by incomplete light harvesting which results in lower current densities. In addition to counteract incomplete light harvesting by employing thicker and highly porous films, organic sensitizers with higher molar extinction coefficients and wider spectral bandwidths have been designed, which are economical as well as environmental friendly [9]. The recent advent of CH_3_NH_3_Pb*X*_3_ (*X* = Cl, Br, I) as a hole conductor or as a sensitizer in solid-state cells, has yielded a photoelectron chemical conversion of 15% [10-13]. However, most of these cells employed mesoporous TiO_2_ nanoparticles for the loading of perovskite thereby offering scope for the cell performance to be further improvised by employing photoanode materials with better porosity and better charge transport characteristics.

Herein we report a photoanode of ssDSC made of one-dimensional electrospun TiO_2_ nanofibers (NF), with additional hierarchical structures to improve the light harvesting without sacrificing the dye attachment. The motivation for this work is to facilitate complete infiltration of spiro-OMeTAD through the large pores prevalent in between the web-like nanofibers and to improve dye loading with the additional hierarchical nanorods grown on the surface of nanofibers. The hierarchical fibrous photoanodes, which are about 4-μm thick, exhibit power conversion efficiency of 2.14%, which to the best of our knowledge, is the highest efficiency in the nanofiber-organic sensitizer-ssDSC system. Also, an organic sensitizer named D358 which has a high molar extinction coefficient of 6.7 × 10^4^ M^-1^ cm^-1^ at *λ*_max_ = 532 nm [14] has been used to sensitize the fibrous photoanodes.

## Methods

The fluorine-doped tin oxide (FTO, <14 Ω/sq, 2.2-mm thick, Pilkington, Solar Energy Technology Co, Ltd, Wuhan Jinge, China) substrates are first etched with Zn powder (Sigma Aldrich, St. Louis, MO, USA) and hydrochloric (HCl) acid (4 M, Sigma Aldrich) to form the desired pattern, which are subsequently cleaned with soap and ethanol (Sigma Aldrich). Then a thin compact layer of TiO_2_ nanoparticles referred to as the blocking layer (approximately 80 nm) is deposited by aerosol spray-pyrolysis at 450°C using ambient air as carrier gas [15]. For spray-pyrolysis, a solution of titanium diisopropoxide bis(acetylacetonate) (Sigma Aldrich, 75 wt.% in isopropanol) and absolute ethanol is used in the ratio 1:9 by volume. For the synthesis of NF, a sol–gel solution comprising 0.8 g PVP (M_w_ = 1,300,000, Aldrich), 4 g titanium(IV) butoxide (97%, Aldrich), 1.18 g acetyl acetone (≥99%, Sigma Aldrich) in 10 mL methanol is prepared and electrospun at 25 kV with a feed rate of 0.3 mL/h using NANON (MECC Co., Brooklyn Center, Hennepin County, MN, USA) electrospinning setup. The nanofibers are collected on the blocking-layer-deposited FTO substrates which are placed on a metallic collecting plate of electrospinning setup. Then the composite mat of nanofibers is calcined at 450°C in a box furnace for 5 h to remove the organic components and to get crystalline TiO_2_ nanofibers. In order to synthesize hierarchical nanofiber (HNF), the substrates with the mat of TiO_2_ nanofibers are immersed in a Teflon-lined autoclave (Fisher Scientific Pte Ltd, UE Tech Park, Singapore) containing a solution of 20 mL HCl, 20 mL deionized (DI) water, and a known amount of titanium isopropoxide (Aldrich). The autoclave is then sealed and put in to a preheated oven at 150°C for reaction times of 0.5, 1, 2, and 3 h.

The nanofibers and hierarchical structures are sensitized with D358 dye (indoline dye, Mitsubishi Paper Mills Limited, Sumida, Tokyo, Japan) by immersing them in the dye solution [0.5 mM, 50% acetonitrile (ACN, Merck & Co, Inc, Whitehouse Station, NJ, USA), 50% tertiary butanol (Sigma Aldrich) and 0.1 M cheno (Sigma)] for 4 h, followed by rinsing in ACN. An organic hole conductor namely spiro-OMeTAD [2,2′,7,7′-tetrakis(*N*,*N*-di-*p*-methoxyphenylamine) 9,9′-spirobifluorene] (Merck KGaA, Darmstadt, Germany) is dissolved in chlorobenzene (Sigma Aldrich) and spin-coated on these substrates. Additives like Li(CF_3_SO_2_)_2_ N (Sigma Aldrich), *tert*-butylpyridine (Sigma Aldrich), and FK102 dopant are added to the above solution [16]. The masked substrates are placed in a thermal evaporator for gold (Au) deposition via shadow masking. The thickness of the Au electrode is about 80 nm, and the active area is defined by the overlapping of TiO_2_ and Au measuring 0.64 cm^2^.

Cross-sectional images are recorded by field emission scanning electron microscope (FESEM, JEOL, JSM-7600 F, 5 kV; JEOL Ltd, Akishima, Tokyo, Japan). The film's thickness is measured using Alpha Step IQ Surface Profiler (KLA Tencor, Milpitas, CA, USA). The phase and crystallographic structure of the nanostructures are characterized by x-ray diffraction (XRD) using a Bruker D8 Advance with Cu K_α_ radiation (Bruker Corporation, Billerica, MA, USA). The structural morphology, phase, and crystallinity are analyzed through selected area electron diffraction (SAED) and high-resolution transmission electron micrographs (HRTEM) using JEOL 2100 F operating at 200 keV. For dye loading experiments, the dye molecules are desorbed by using TMAH (0.1 M, Sigma Aldrich) solution and the resultant solutions are inspected via UV–vis-NIR spectrophotometer (UV3600, Shimadzu Co Ltd, Beijing, China) with 282-nm wavelength light source. Photocurrent-voltage measurements are taken using San-EI Electric, XEC-301S (San-EI Electric Co, Ltd, Higashi-Yodogawa, Osaka, Japan) under AM 1.5 G. Incident photon to current conversion efficiency (IPCE) is determined using PVE300 (Bentham Instruments Ltd, Reading, Berkshire, UK), with dual xenon/quartz halogen light source, measured in DC mode and no bias light is used. Electrochemical impedance spectroscopy measurements are recorded using AutoLab PGSTAT302N (Metrohm Autolab BV, Utrecht, The Netherlands) under illumination condition, and different bias potentials are applied ranging from 0.5 V to open circuit voltage. An alternating sinusoidal signal of 10 mV and frequency ranging from 100 KHz to 0.1 Hz are used.

## Results and discussion

**Figure 1:**
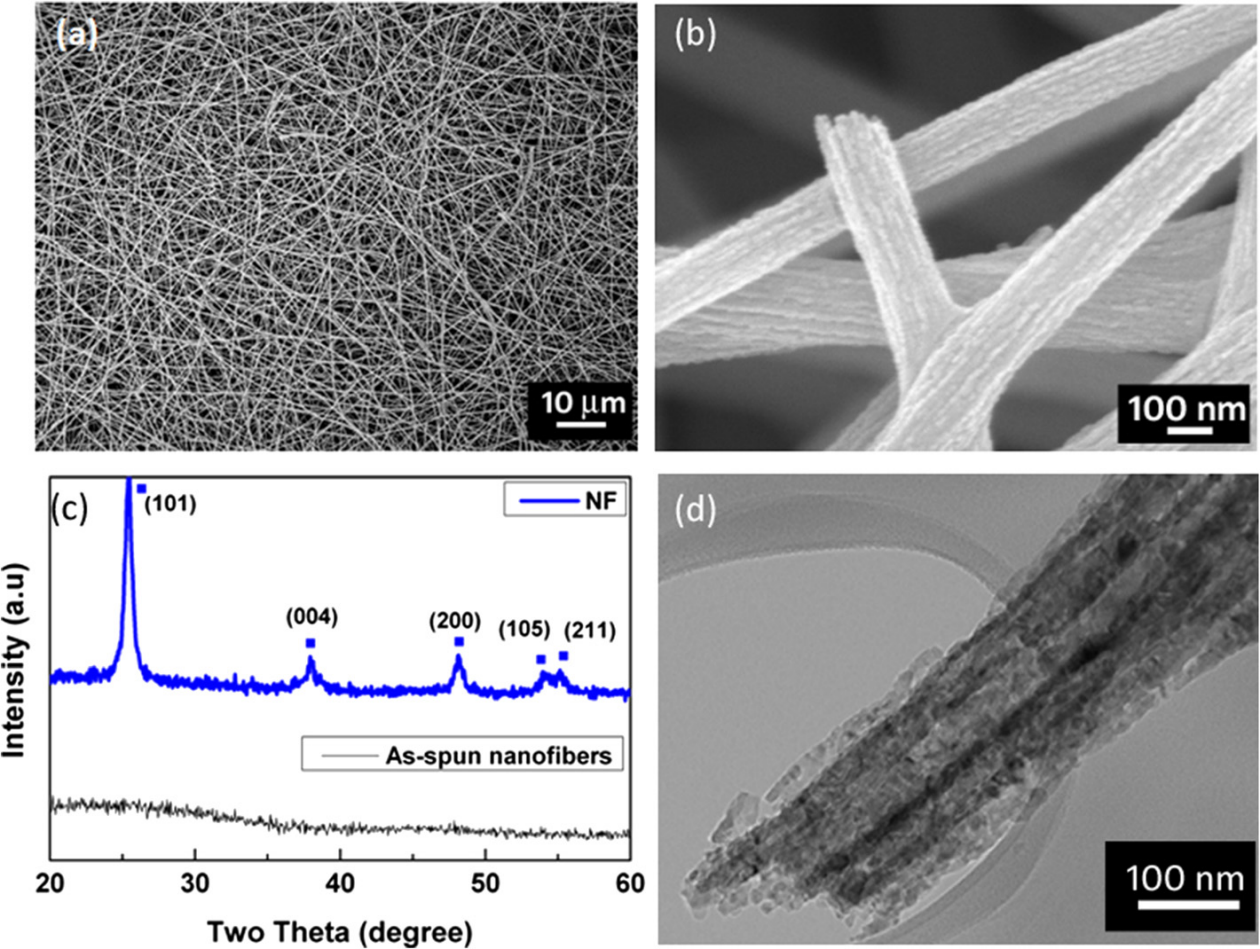
**Images and XRD spectra of TiO**_**2 **_**nanofibers.** FESEM images of the calcined TiO_2_ nanofibers on FTO substrate **(a)** low magnification and **(b)** high magnification. **(c)** XRD spectra of as-spun nanofibers and calcined nanofibers (NF). Blue solid squares denote anatase phase. **(d)** TEM image of the as-spun nanofibers.

Figure 1a shows the FESEM image of the nanofibers after sintering at 450°C, a step necessary to remove polymer and other organic solvents and to yield the anatase phase of the nanofibers. The diameter of these nanofibers is in the range of 150 to 200 nm. The SEM image clearly reveals long, interconnected, and web-like network with voids in between each fiber. The interconnected nanofibers form a mesh-like morphology, which is beneficial for percolation of viscous fluids or polymers. Figure 1b shows a high-resolution FESEM image of a single strand of manually broken nanofiber. The broken end of the nanofiber reveals that it is not hollow but is composed of internal nanostructures called nanofibrils [17,18]. A crack-free surface can be clearly observed. Figure 1c shows the XRD spectra of the nanofibers before and after calcination. The as-spun nanofibers are amorphous in nature. The polycrystalline nature of the nanofibers is revealed after calcination at 450°C. The diffraction peaks for the NF sample can be indexed to the anatase phase of TiO_2_ (JCPDS no 21–1272). Figure 1d shows the low-magnification TEM image of TiO_2_ nanofiber after calcination. The surface of the nanofiber appears to be defect free. The dark areas result from the varying crystalline density which is due to the presence of nanofibrils within each nanofiber. The formation of such structures is explained in our previous work [17]. The broken edges of the nanofibers arise during the sample preparation for TEM.

**Figure 2:**
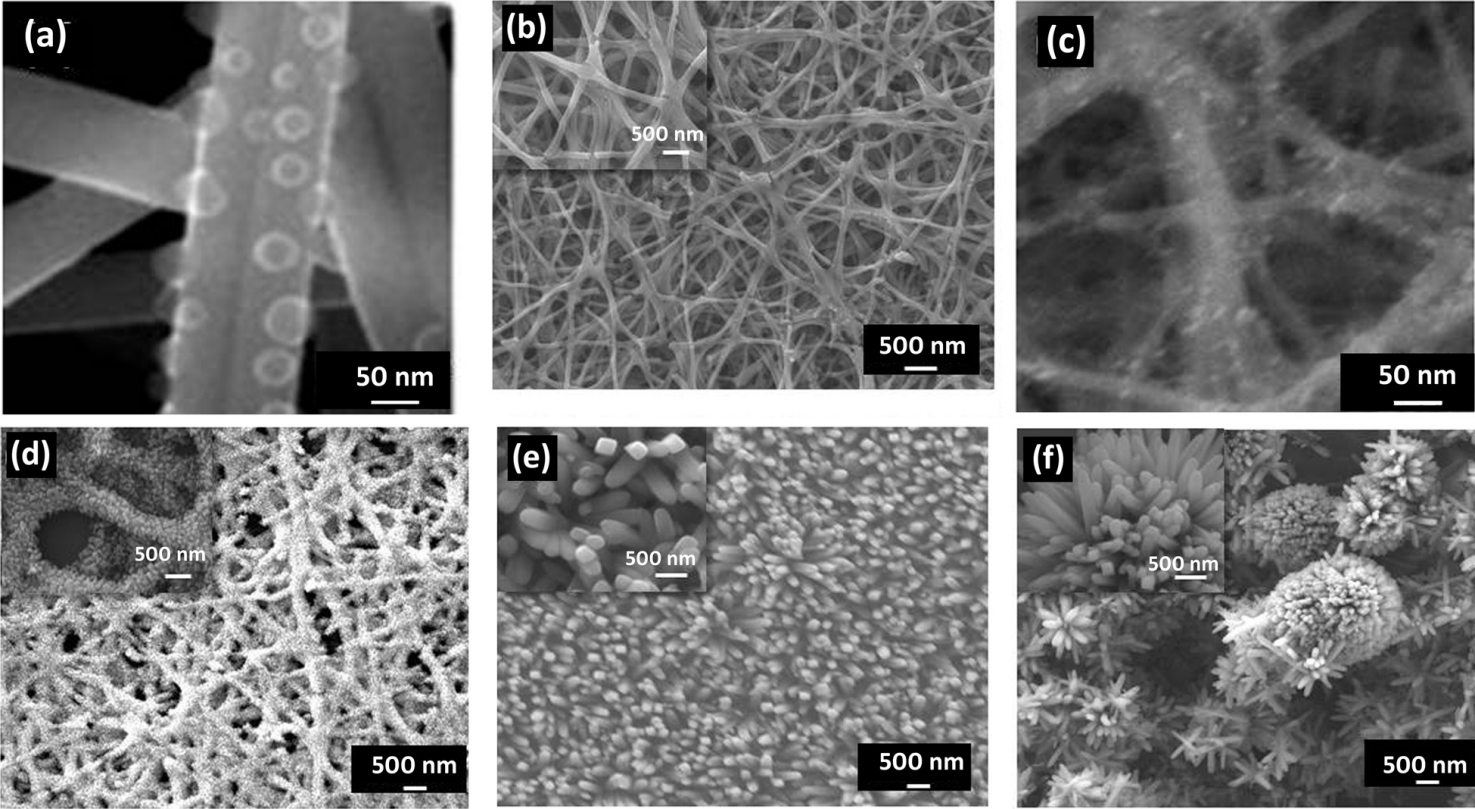
**FESEM images of the secondary growth on TiO**_**2 **_**nanofibers at different reaction time. (a)** 10 min, **(b)** 30 min, **(c)** 45 min, **(d)** 1 h, **(e)** 2 h, and **(f)** 3 h. Insets show the magnified images of nanostructures.

With the objective of facilitating higher dye loading, the nanofiber scaffold is subjected to hydrothermal treatment to grow secondary structures on the surface of the nanofibers. We try to investigate the effect of reaction time on hydrothermal reaction and observe the morphology of the nanofibers. This study will also help in understanding the formation mechanism of such nanostructures. As shown in Figure 2, the nanofibers prepared using different reaction times exhibit varying surface morphologies. Figure 2a shows small nuclei centers on the nanofibers after 10 min of reaction time. These centers will act as the core from which the rod-like nanostructures will grow. Figure 2b shows the nanofibers which are subjected to hydrothermal treatment at 30 min. No growth of secondary structures is observed here. The diameter of the nanofibers is in the range of 150 to 200 nm. A close inspection of the FESEM image (inset of Figure 2b) reveals that the nanofibers have rough surface, which is instrumental in the growth of hierarchical nanostructures. The surface roughness leads to reduction in energy barrier for heterogeneous nucleation of nanostructures and thus aids further growth. In the present case, different size nanorods grow preferentially on the rough nanofibers. With prolonged reaction time to 45 min, the spherical morphology tends to form irregular aggregates (Figure 2c). Figure 2d shows that for the reaction time of 1 h, secondary growth looks like nanorods covering approximately 80% of the nanofiber surface area. These nanorod-nanofiber structures are designated as HNFs throughout this paper. The average diameter of HNF is in the range of 500 to 700 nm. These nanorods not only increase the diameter of the nanostructure but also make its surface coarse. With further increase in reaction time to 2 h, the density, length, and width of the secondary structures on the nanofiber scaffold increase to a greater extent as shown in Figure 2e, leading to the filling of pores between each fiber. These nanostructures appear nucleated from the nanofibers and spread outwards. From the inset image of Figure 2e, it can be observed that the small nanostructures are of tetragonal shape, with the tip having a morphology which is close to the square facets. The diagonal size of the tetragonal nanorod measures about 200 to 250 nm. For 3-h reaction time, the nanofiber morphology gives way to the flower-like nanostructures (Figure 2f). The growth of the flower-like nanostructures occurs at the expense of the seeding layer, which in this case is the nanofiber scaffold. This leads to complete dissolution of the nanofiber network. The diameter of flower-like nanostructures is approximately 240 to 280 nm. As the nanorods grow in size their tips become more tapered. It is clear that the length, diameter, and density of the secondary structures can be tuned by varying the reaction time during the hydrothermal growth. Since a porous network of nanofibers will aid easy and complete infiltration of HTM layer, HNF synthesized for a hydrothermal reaction time of 1 h are apt for solar cell application. These synthesized nanostructures are believed to not only retain the porous network but also display higher anchoring sites for the dye molecules, thereby leading to increased light harvesting.

Based on the time-dependent study, a growth mechanism can be proposed for these nanostructures. In the initial stage, the reacting solution consists of Cl^-^ ions and Ti precursors. Cl^-^ ions diffuse out leading to nucleation of Ti precursor on the surface of nanofibers. These precursors tend to settle on the nanofibers surface and act as nuclei for further growth. It is through Ostwald’s ripening process that the initially formed aggregates gradually scavenge, accompanied by the growth of rod-like nanostructures. It is reported that the ratio of Cl^-^ ions to Ti in the solution is important [19,20]. The high acidity and low concentration of Cl^-^ ions favor the growth of rutile-phase rod-like nanostructures. The precursor containing HCl as the acid medium has a tendency to form rod-shaped rutile TiO_2_ nanostructures. It appears that the anatase nucleates and grows first according to Ostwald’s law, and rutile nuclei appear eventually when the successive reaction is taking place since rutile is thermodynamically the most stable phase [20]. Thereafter, the rutile quickly grows epitaxially at the expense of mother anatase crystallites via a dissolution and precipitation process [21]. Both rutile and anatase belong to the tetragonal crystal system, consisting of TiO_6_ octahedra as a fundamental structural unit. Their crystalline structures differ in the assembly of the octahedral chains [22,23]. Rutile has 42 screw-axes along the crystallographic *c*-axis. The screw structure promotes crystal growth along this direction, resulting in a crystal morphology dominated by the {110} faces [24]. Therefore, rutile nanoparticles are usually rod-like.

**Figure 3:**
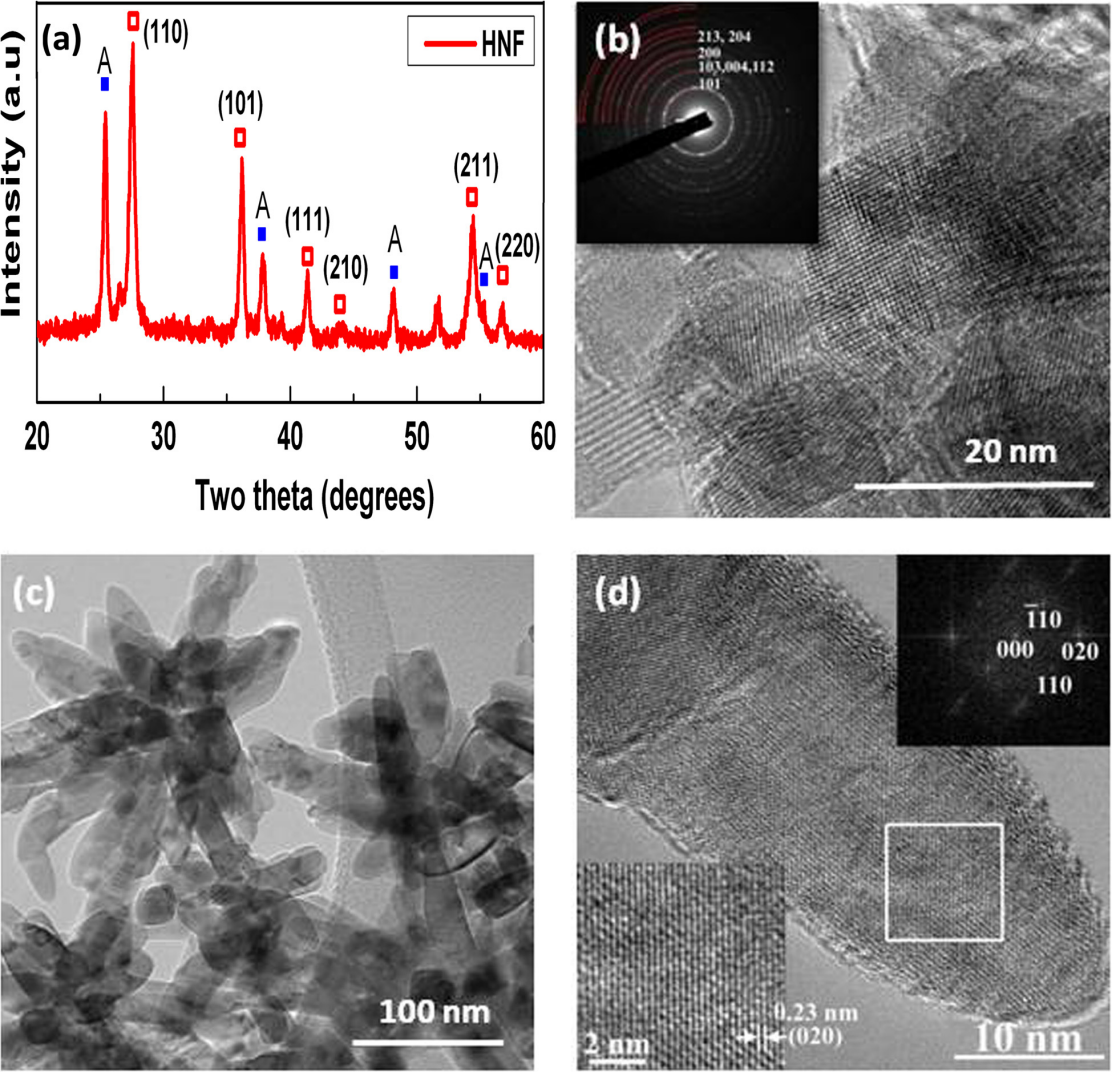
**XRD spectrum, HRTEM and TEM images of nanofibers and their secondary growth. (a)** XRD spectrum of nanofibers after hydrothermal treatment to form HNF. The additional red hollow squares denote rutile phase. **(b)** HRTEM image of as-spun nanofibers showing polycrystallinity. **(c)** TEM and **(d)** HRTEM images of the secondary growth on nanofibers. Insets show the SAED patterns for both the samples.

Figure 3a shows the XRD spectrum of HNF sample taken after hydrothermal treatment on nanofibers (1 h at 150°C). HNF is composed of both anatase (JCPDS no 21–1272) and rutile phase (JCPDS no 21–1276), and the weight percentage of each phase is given in Table 1. The sharp diffraction peaks of the NF and HNF samples point to their highly crystalline nature, which is necessary for good electron transport. To better understand the structure of TiO_2_ nanofibers and hierarchical structures, TEM/HRTEM measurements are taken to study the samples. In the HRTEM image (Figure 3b), the distance between the adjacent lattice fringes is 0.35 nm. The SAED pattern (inset of Figure 3b) confirms that the nanofibers are polycrystalline in nature and posses anatase phase. This evaluation is consistent with the XRD analysis. Figure 3c shows low magnification TEM image of secondary nanostructures grown on TiO_2_ nanofibers with a reaction time of 1 h. The surface of the nanofibers is completely covered with many nanorod-like structures. The HNF nanostructures appear discontinuous due to the breakage of the nanofibers during sample preparation. It is evident that the nanorods grow at the expense of the nanofibers as the diameter of the electrospun nanofiber is not visible in the TEM image. These nanorods are not growing perpendicular to the nanofiber surface but are inclined at an angle. Also, the nanorods are found to be anchored to the nanofibers effectively with large-area connection. The nanorods grow heterogeneously all over and cover most of the nanofiber surface. From HRTEM image of a single nanorod (Figure 3d), the lattice fringes with interplanar spacing is observed to be approximately 0.23 nm, which can be indexed to the tetragonal rutile TiO_2_ phase (JCPDS no. 21–1276). The corresponding SAED pattern recorded from the same area (inset of Figure 3d) demonstrates that the secondary nanorods are single crystalline in nature and exist in pure rutile phase. From the combined data of XRD and HRTEM, it can be inferred that the secondary nanostructures on nanofibers are single crystalline with a preferred [110] orientation. Thus, it can be concluded that the hydrothermal reaction in strong acidic aqueous environment produces well-crystallized rutile secondary nanostructures on TiO_2_ nanofibers.

**Table 1:** Physical properties and photovoltaic parameters of plain nanofiber and hierarchical nanofiber-based DSCs

**Electrode**	**Anatase (%)**	**Rutile (%)**	**Crystallite size (nm)**	**Dye loading (×10**^**-8**^ **mol/cm**^**2**^**)**	***J***_**sc **_**(mA/cm**^**2**^**)**	***V***_**oc **_**(V)**	**FF (%)**	***η *** **(%)**
NF	100	0	16.1	4.25	3.93	0.84	0.43	1.42
HNF	25.31	68.37	26.7	6.0	4.05	0.92	0.58	2.14

**Figure 4:**
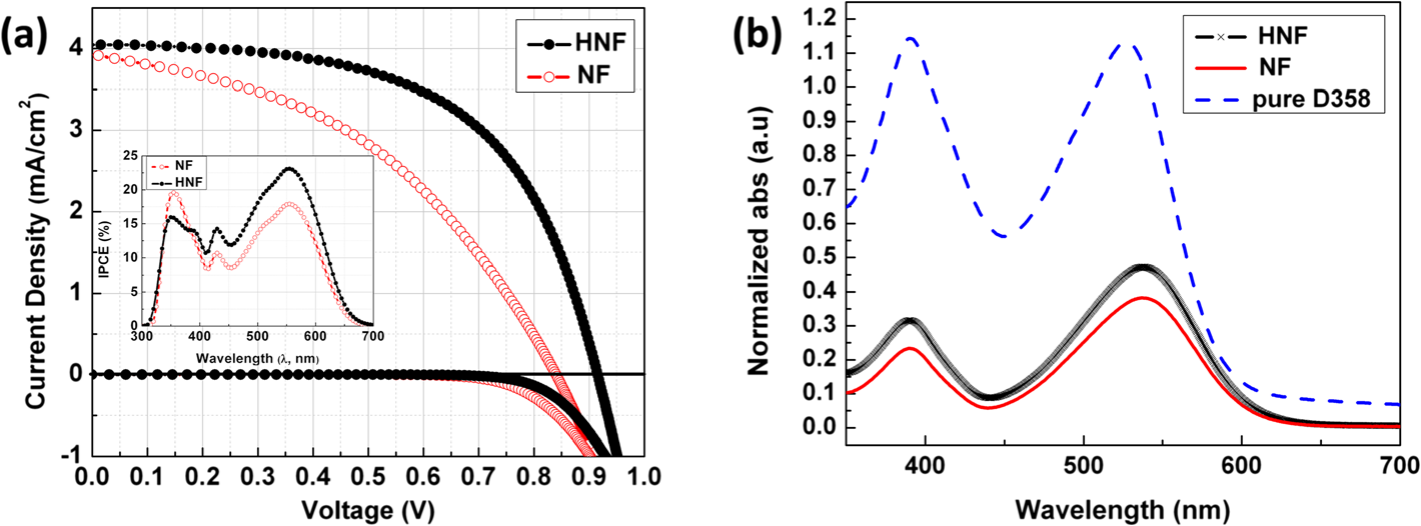
**Photocurrent density-voltage curves and UV–vis spectra of NF- and HNF-based ssDSC. (a)** Photocurrent density-voltage characteristics of NF- and HNF-based ssDSC measured under one sun illumination. IPCE spectra of the above-mentioned cells are given in the inset. **(b)** UV–vis absorption spectra of the amount of dye desorbed from the respective photoanodes.

The calcined nanofibers and nanofibers with secondary nanostructures are employed as photoanodes in ssDSC. The thicknesses of the photoanodes are about 4 μm. The current densities vs. voltage curves for the fabricated ssDSC are shown in Figure 4a and the cell parameters are summarized in Table 1. IPCE spectra are also recorded to better understand the performance of ssDSC (inset of Figure 4a). The HNFs comprise anatase and rutile phases (Table 1; the calculations are given in Additional file 1), and it is well established in literature [25-27] that DSCs fabricated using a mixture of anatase and rutile phases exhibit improved cell performance as compared to those of pure anatase phase. Hence, the synthesized HNF are believed to perform better. The HNF-based photovoltaic cells always outperformed the NF-based photovoltaic cells for various photoanode film thickness (Additional file 1: Table S1). This enhanced photovoltaic performance can be attributed to increased current density (*J*_sc_*)*, open circuit voltage (*V*_oc_), and fill-factor (FF). The rutile nanorods on anatase nanofibers provide additional dye anchoring sites, which is significant for generating high *J*_sc_ (inset of Figure 4a). The higher dye loading capability of the HNF is validated using UV–vis spectroscopy (Figure 4b). The amount of dye loaded on HNF is approximately 6.0 × 10^-8^ mol/cm^2^, which is 41.17% higher than the amount of dye adsorbed on NF (approximately 4.25 × 10^-8^ mol/cm^2^). Thus, the absorbance of dye on HNF photoanode is larger than the NF-based photoanode as seen in Figure 4b. The presence of more number of dye molecules in case of HNF clearly suggests that the nanorods impart higher surface area and thus are beneficial in improving light harvesting by generating more photoelectrons. This correlates well with the high IPCE observed in case of HNF cell. The dip in IPCE at 340 to 385 nm for the HNF cell had negligible contribution to the short-circuit current density as the solar photon flux in this wavelength is low. Thus, the short-circuit current density integrated from IPCE spectra is higher for the HNF-based cell with respect to that of the NF solar cell.

**Figure 5:**
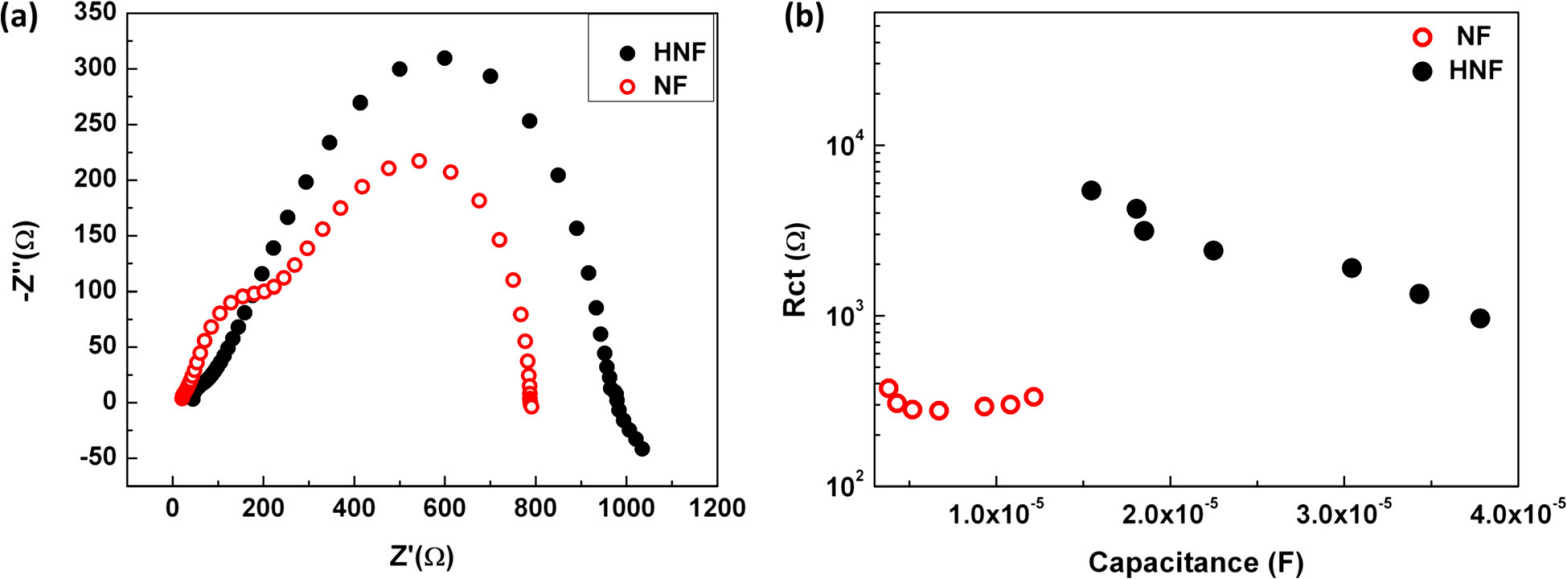
**Nyquist plots for HNF and NF cells. (a)** Nyquist plots of impedance data for HNF and NF cells. **(b)** Charge recombination resistance vs. chemical capacitance.

The electrochemical impedance spectroscopy measurements are further performed to elucidate the enhancement of *V*_oc_ in the HNF cell. Figure 5a depicts the Nyquist plots of the two cells under open circuit voltage condition. The line connecting the first semicircle at higher frequencies and the semicircle at intermediate frequencies denote the charge transport resistance within the TiO_2_ film. By fitting the EIS spectra using the transmission line model of DSC [28,29], it is observed that the resistance to transport of charge within plain nanofiber is higher because the charge has to encounter more number of grain boundaries as each nanofiber is composed of several nanofibrils. Whereas in the case of HNF cell, each nanofiber is covered with single crystalline nanorods in which the transport of electron is less inhibited. Since the nanofiber acts as a seeding layer for the growth of nanorods and the nanorods grow at the expense of the nanofiber, the nanofiber is reduced in size leading to less number of defects (as in Figure 3c). The second semicircle at the intermediate frequencies in the Nyquist plot denotes the charge recombination resistance between TiO_2_ and HTM layer. It is observed that the semicircle of HNF cell is larger than the semicircle of the NF cell, implying that the HNF cell exhibited higher resistance to charge recombination as compared to that of NF cell. The plot of charge recombination resistance (Rct) vs. chemical capacitance (Cμ) is shown in Figure 5b. It was reported that this approach provides information analogous to that obtained from the approach of comparing lifetimes or dark current at constant charge density [30]. So at a particular Cμ which is a measure of density of states at quasi-Fermi level, Rct of HNF is higher than the Rct of NF (Figure 5b). This indicates that the HNF exhibited higher resistance to recombination of injected charge with holes in spiro-OMeTAD. As a result of the higher charge recombination resistance, HNF cell exhibited higher *V*_oc_. The densely populated nanorods with higher dye loading provide greater screening between the injected electrons in TiO_2_ film and holes in HTM, thereby suppressing the recombination of electrons at the TiO_2_ and spiro-OMeTAD interface [31]. In the case of NF-based cell, the pores between the nanofibers are big and the dye coverage is relatively lower, ensuing the recombination of electron hole pair at TiO_2_/spiro-OMeTAD. This is also supported by the delayed onset of dark current in the case of HNF-based DSC, which is suggestive of the good blocking property of HNF (as seen in Figure 4a).

## Conclusions

A simple, effective, and economical approach to improve the light harvesting of electrospun nanofibers has been reported in this work. By employing hydrothermal route, nanorods are grown on electrospun nanofibers. The resulting TiO_2_ nanostructures consist of both anatase and rutile phases. The secondary growth of nanorods is in [110] orientation and are single crystalline in nature, a characteristic which plays a significant role in reducing the charge transport resistance throughout the film. Upon integration of the synthesized nanostructures as photoanodes for solid-state dye-sensitized solar cells, the hierarchical nanofibers exhibit 2.14% efficiency with *J*_sc_ and *V*_oc_ values being 4.05 mA/cm^2^ and 0.92 V, respectively. The nanorods provide additional surface area for dye loading, which helps to improve the light harvesting of the fibers by 41%. In addition to dye adsorption, the presence of larger number and densely packed dye molecules offers greater extent of screening between the electrons injected into the TiO_2_ conduction band and holes in spiro-OMeTAD. Owing to their crystallinity and packing density, the hierarchical nanofibers exhibit superior properties as compared to the plain nanofibers for solar cell application. These nanostructures can also be employed in fuel cells or in water splitting applications, where high surface area is required with efficient transport in 1D nanostructures. Furthermore, the combination of hierarchical nanofibers with CH_3_NH_3_PbI_3_, as a sensitizer with high absorption coefficient, can lead to inexpensive yet high efficiency solid-state cells [32].

## Competing interests

The authors declare no competing interests.

## Authors’ contributions

DS and SA conceived the idea of the project and carried out the characterization measurements. DS synthesized the nanofibers and fabricated the devices. SA performed the hierarchical growth. SSP contributed to the TEM and SAED characterizations. SGM supervised the project. All authors read and approved the final manuscript.

## Authors’ information

DS is currently doing her Ph.D. in Materials Science Engineering at Nanyang Technological University, Singapore. She did her M.Sc. in Advanced Materials (Nanotechnology) studies at University Ulm, Germany and her B.Tech. in Metallurgical and Materials Engineering at IIT-Roorkee. Currently, her research focus is on electrospinning organic/inorganic nanostructures and investigate their properties for solar energy application.

SA obtained her Ph.D. in electronics engineering in 2011 from National University of Singapore (NUS) on nanostructured materials for dye-sensitized solar cells. Currently, she is a research fellow under SM at Energy Research Institute (ERI@N), Nanyang Technological University (NTU). Her research is aimed at new synthesis pathways for porous inorganic nano-materials and perovskite materials for solar energy applications.

SSP obtained his Ph.D. in Materials Science and Engineering in 2011 from Nanyang Technological University on novel intermediate temperature solid oxide fuel cell (SOFC) apatite electrolyte. Currently, he is a postdoctoral research associate working with Professor Mary Ryan and Dr. Stephen Skinner at Imperial College London. His research is aimed at surface chemistry and structure of mixed ionic-electronic conducting layered perovskite for SOFC cathode.

SGM is a professor in the School of Materials Science & Engineering at the Nanyang Technological University, Singapore. At NTU, he also holds the post of Executive-Director, Energy Research Institute at NTU (ERI@N). Prior to joining NTU in 2001, Subodh has over 10 years of research and engineering experience in the microelectronics industry where he held senior managerial positions in STATS Singapore, National Semiconductor, and SIMTech. His main areas of research comprise printed electronics, sensors, photovoltaics, and supercapacitors and batteries. Common to all these projects are methods of solution processing of semiconductors (organic, carbon nanotubes, or inorganic nanowires), fundamental device physics studies, and device integration. For his work in organic thin-film transistors, SM and his team recently won the IEEE 2008 George E. Smith Award. He is also the recipient of Ohio State University’s Professional Achievement Award in 2012. Major research projects include Competitive Research Program Funding from the National Research Foundation on ‘Nanonets: New Materials & Devices for Integrated Energy Harnessing & Storage,’ Polymer & Molecular Electronics with A*STAR, and a DARPA-funded program on printed charge storage devices. SM has published more than 250 research papers and has active collaborations with UCLA, Northwestern University, CEA/CNRS France, IIT-Bombay, NUS, and local research institutes. SM received his Bachelors’ degree from IIT-Bombay and his M.S./Ph.D. degrees from The Ohio State University.

## Supplementary Material

Additional file 1: Figure S1 X-ray diffraction pattern from which the weight percentage of each phase was calculated. **Table S1:** Effect of photoanode thickness on photovoltaic parameters of plain nanofiber and hierarchical nanofiber-based DSCs respectively. Click here for file
