# Supplementary material for: A maskless synthesis of TiO2-nanofiber-based hierarchical structures for solid-state dye-sensitized solar cells with improved performance
Source: Nanoscale Res Lett. 2014 Jan 10;9(1):14. doi: 10.1186/1556-276X-9-14 (PMC3895802; doi:10.1186/1556-276X-9-14)
Supplement: Additional file 1: Figure S1 — X-ray diffraction pattern from which the weight percentage of each phase was calculated. Table S1: Effect of photoanode thickness on photovoltaic parameters of plain nanofiber and hierarchical nanofiber-based DSCs respectively. [file 1556-276X-9-14-S1.docx]

**Supporting Info**

**Figure Legends**

Fig S1: X-ray diffraction pattern from which the weight percentage of each phase was calculated.

**
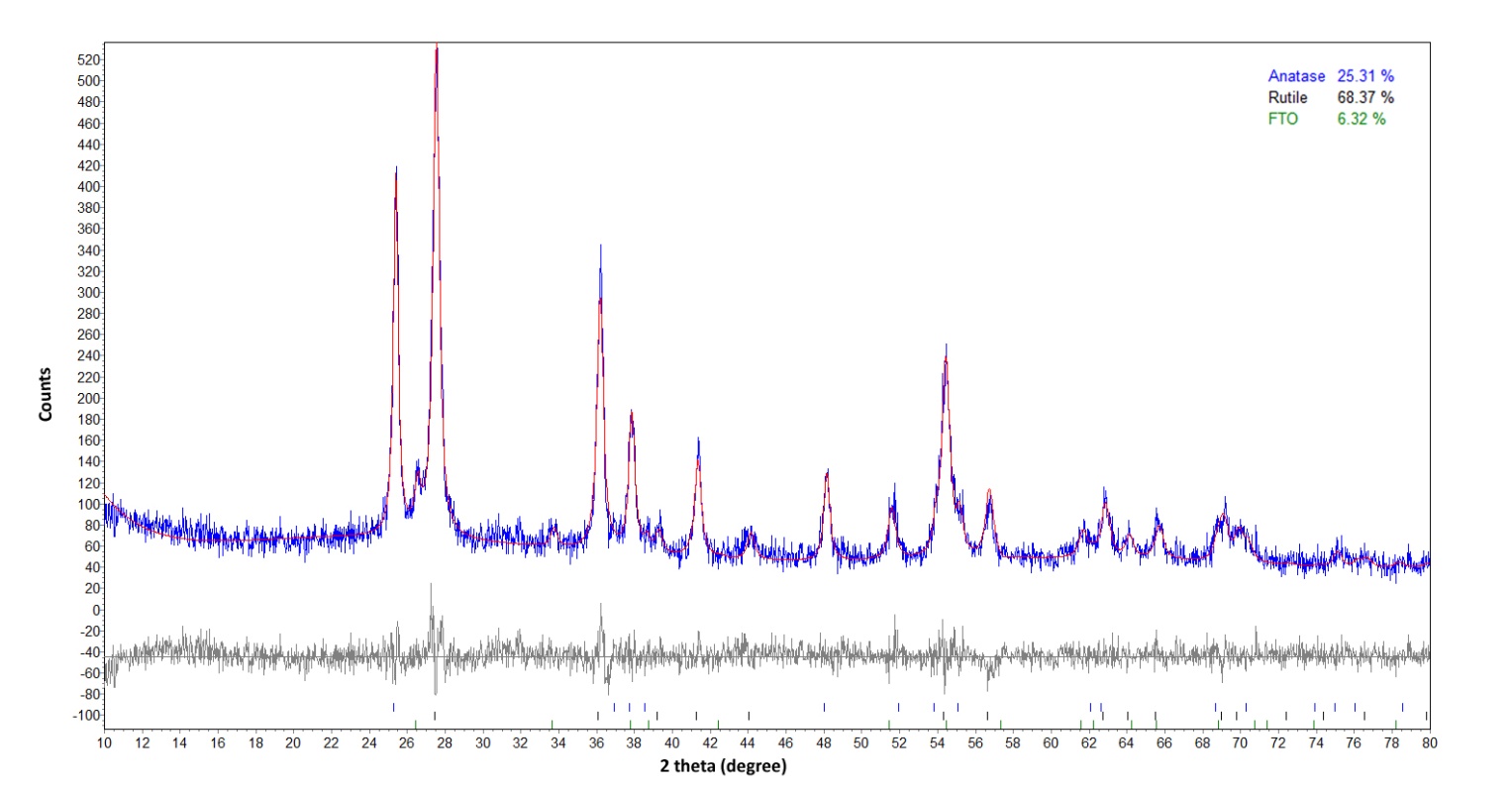
**

**Fig S1**

The starting Rietveld refinement model used CIF files of anatase [ICSD 9852] and rutile [ICSD 51940]. The fundamental parameters peak-shape profile was used; a five-coefficient Chebychev polynomial and 1/*x* background, zero error, scale factors, unit cell parameters and crystal size were sequentially refined using the TOPAS V3 program. The weight percentage of each phase was calculated based on quantitative phase analysis using the Rietveld method [[1](#_ENREF_1), [2](#_ENREF_2)]:

| $W_{\alpha}=\frac{S_{\alpha}(ZMV)_{\alpha}}{\sum_{i} (S_{i}(ZMV)_{i})}$ | (1) |
| --- | --- |

where W_α_= is the weight fraction of phase α, *S* = Rietveld scale factor, *Z* = No. of formula units in unit cell, *M* = molecular mass of formula unit and *V* = unit cell volume.

Table S1: Effect of photoanode thickness on photovoltaic parameters of plain nanofiber and hierarchical nanofiber based DSCs respectively.

| Electrode | Jsc (mA/cm^2^) | Voc (V) | FF (%) | η (%) |
| --- | --- | --- | --- | --- |
| NF-1.6 µm | 0.46 | 0.96 | 0.49 | 0.22 |
| 4 µm | 3.93 | 0.84 | 0.43 | 1.42 |
| 6.5 µm | 3.41 | 0.84 | 0.46 | 1.31 |
| HNF-1.6 µm | 1.06 | 0.91 | 0.72 | 0.69 |
| 4 µm | 4.05 | 0.92 | 0.58 | 2.14 |
| 6.5 µm | 3.34 | 0.87 | 0.54 | 1.58 |

**Table S1**

The photoanode film thickness is determined by increasing the density of nanofibers which is achieved by increasing the electrospinning time. With increasing film thickness (>4 µm), the pores between the nanofibers become smaller due to overlap of the nanofibers [[3](#_ENREF_3)]. As a result, in case of 6.5 µm HNF, the additional nanorods reduced the pores between the web-like nanofibers thereby reducing the dye loading. Thus for 6.5 µm HNF, Jsc is observed to be lower than that of 6.5 µm NF solar cell. So 4 µm was the optimized thickness for the HNF based solar cell. Also with increasing film thickness, open circuit voltage decreases due to enhanced charge recombination [[4](#_ENREF_4)]. This trend has been observed even in HNF case.

References

1. Rietveld H: **A profile refinement method for nuclear and magnetic structures.** *Journal of Applied Crystallography* 1969, **2:**65-71.

2. Hill RJ, Howard CJ: **Quantitative phase analysis from neutron powder diffraction data using the Rietveld method.** *Journal of Applied Crystallography* 1987, **20:**467-474.

3. Sabba D, Kumar HM, Yantara N, Pham TTT, Park N-G, Gratzel M, Mhaisalkar SG, Mathews N, Boix PP: **High efficiency electrospun TiO_2_ nanofiber based hybrid organic-inorganic perovskite solar cell.** *Nanoscale* 2013.

4. Ito S, Zakeeruddin SM, Humphry-Baker R, Liska P, Charvet R, Comte P, Nazeeruddin MK, Péchy P, Takata M, Miura H, et al: **High-Efficiency Organic-Dye- Sensitized Solar Cells Controlled by Nanocrystalline-TiO_2_ Electrode Thickness.** *Advanced Materials* 2006, **18:**1202-1205.
